# Study on Hydro-Mechanical Coupling Failure and Permeability Enhancement Mechanisms for Sandstone with T-Shaped Fractures

**DOI:** 10.3390/ma16083118

**Published:** 2023-04-15

**Authors:** Ying Zhang, Kun Bi, Jiliang Pan, Xun Xi, Dongsheng Zhang, Shengjun Miao, Meifeng Cai

**Affiliations:** 1 School of Civil and Resource Engineering, University of Science and Technology Beijing, Beijing 100083, China; 2 Shunde Innovation School, University of Science and Technology Beijing, Foshan 528399, China; 3 Shangyuquan Coal Mine, Shanxi Luneng Hequ Electric Coal Development Co., Ltd., Xinzhou 034000, China

**Keywords:** reservoir sandstone, hydro-mechanical coupling, progressive failure, permeability, RFPA2D-FLOW

## Abstract

The rise in the connectivity of the fractures is a key task in oil/gas and geothermal exploitation systems. Natural fractures widely exist in underground reservoir sandstone, while the mechanical behavior of rock with fractures subjected to hydro-mechanical coupling loads is far from clear. This paper employed comprehensive experiments and numerical simulations to investigate the failure mechanism and permeability law for sandstone specimens with T-shaped faces subjected to hydro-mechanical coupling loads. The effects of crack closure stress, crack initiation stress, strength, and axial strain stiffness of the specimens under different fracture inclination angles are discussed, and the evolution processes of permeability are obtained. The results show that secondary fractures are created around the pre-existing T-shaped fractures through tensile, shear, or mixed modes. The fracture network causes an increase in the permeability of the specimen. T-shaped fractures have a more significant effect on the strength of the specimens than water. The peak strengths of T-shaped specimens decreased by 34.89%, 33.79%, 46.09%, 39.32%, 47.23%, 42.76%, and 36.02%, respectively, compared with intact specimen without water pressure. With the increase in deviatoric stress, the permeability of T-shaped sandstone specimens decreases first, then increases, reaching its maximum value when macroscopic fractures are formed, after which the stress suddenly decreases. When the prefabricated T-shaped fracture angle is 75°, the corresponding permeability of the sample at failure is maximum, with a value of 15.84 × 10^−16^ m^2^. The failure process of the rock is reproduced through numerical simulations, in which the influence of damage and macroscopic fractures on permeability is discussed.

## 1. Introduction

Hydro-mechanical coupling modeling of rock in oil/gas and geothermal engineering has been one of the research hotspots in recent years [1,2,3,4]. In the exploration and development of oil and gas, studying the mechanical and permeability properties of reservoir rock under hydro-mechanical coupling can help researchers determine the strength, stability, transport capacity, and reserves of petroleum and natural gas in underground rocks, which is crucial for oil and gas exploration and production. In oil and gas well design, parameters such as permeability, porosity, and permeability of the reservoir need to be considered. Constructing a hydro-mechanical coupling model can simulate the deformation of surrounding formations and the changes in water pressure around oil and gas wells, thereby determining the optimal design. In geothermal energy development, by measuring the permeability distribution of underground rocks, the optimal geothermal energy development scheme can be determined, and the sustainability and stability of the geothermal reservoir can be predicted. Furthermore, studying a hydro-mechanical coupling model can grasp the connectivity of rock fractures and deformation laws, as well as the migration direction and velocity of water, thereby optimizing fracturing design and increasing the permeability of rock reservoirs for increased production [5,6,7,8,9,10]. Currently, both sandstone and carbonate reservoirs have natural fractures and secondary fractures generated by mining disturbances, which can be controlled by coupling water injection and rock pressure to efficiently activate natural fractures and induce secondary fractures, greatly increase rock permeability, and improve the efficiency of oil and gas and geothermal production [11,12]. Many researchers believe that the porosity and damage fractures of reservoir rocks have an important impact on the movement of fluids in the rock, and the presence of fluids will also have a significant impact on the occurrence conditions and mechanical properties of the rock [13,14]. In addition, the study also believes that the size and distribution of the stress field will directly affect the state of micro-cracks inside the rock, change the permeability inside the rock mass, and then affect the effective stress distribution inside the rock mass. The permeability of rock changes significantly after the rock reaches the yield stress [15,16,17,18,19,20,21]. The stress field and seepage field of the rock will affect each other. The increase in stress on the rock will increase the porosity of the rock, thereby increasing its permeability and seepage pressure of the rock. At the same time, the change in seepage pressure will affect the effective stress of the rock. Therefore, studying the hydro-mechanical coupling process and evolution mechanism of rocks is very beneficial to the development of oil and gas and geothermal engineering.

At present, researchers have carried out a lot of research on the hydro-mechanical coupling process of rocks. Baud et al. investigated the mechanical properties of different types of dry and water-saturated sandstones and explored the weakening effect of water [22]. The studies of Lajtai et al. and Masuda also showed the weakening effect of water on the strength and deformation of rocks [23,24]. Helland and Raab, Wang and Park, and Wang et al. considered that the permeability of rocks is as important as the mechanical properties under hydro-mechanical coupling conditions [25,26,27]. They investigated the permeability evolution law during the complete stress-strain process and showed that the permeability evolution exhibits stages during the stress-strain process. Zhu and Wong, David et al. investigated the relationship between permeability and porosity in porous rocks, the effect of stress on permeability, and damage modes [28,29]. Chen et al. conducted a triaxial compression experiment of hydro-mechanical coupling on Beishan granite and proposed an empirical upper bound permeability model based on the relationship between microstructure and macroscopic permeability [30]. Xiao et al. conducted hydro-mechanical coupling experiments under different seepage pressures and established a piecewise functional relationship model between permeability and stress according to the change law of permeability [31]. In order to study the strength and failure mechanism of fractured rock under seepage pressure, Lin et al. artificially prefabricated sandstone specimens with different fracture inclination angles, and established a uniaxial seepage-stress loading device, which was combined with an acoustic emission (AE) system [32]. In addition, uniaxial compression tests were performed with and without seepage pressures. Zhou et al. conducted conventional triaxial compression (CTC) and seepage-stress coupling (HM) tests on Beishan granite and used an acoustic emission (AE) spatial positioning system to monitor the whole process of rock progressive failure [33]. Under the coupling, the crack closure stress disappears, and the crack initiation stress is higher than that of the CTC test. When the damage stress is reached, the pore pressure can promote crack development. References carried out research on the effect of hydro-mechanical coupling on the physical and mechanical properties of rocks [34,35,36].

In recent years, with the continuous development of numerical analysis technology, many scholars have applied discrete element (DEM) or finite element methods (FEM) to numerically analyze the hydro-mechanical coupling problem of fractured rocks, which can better characterize the damage, failure, penetration, block sliding, and other processes of fractured rocks under the hydro-mechanical coupling [37,38,39,40]. For example, Yuan and Harrison developed a hydro-mechanical coupling constitutive model including strength and stiffness degradation and used it to study the progressive damage and flow behavior of heterogeneous rocks [37]. Cai et al. used particle flow code 2D 6.0(PFC 2D) software to simulate the triaxial compression test under hydro-mechanical coupling, studied the mechanical characteristics of sandstone, pore pressure distribution, and permeability changes, and revealed the changes in mesostructure, cumulative damage, and permeability evolution synergistic effect [38]. Chen et al. used two-dimensional realistic failure process analysis-flow (RFPA2D-FLOW V2) code software to simulate the hydro-mechanical coupling test of sandstone under triaxial compression, analyze the failure process and failure mode of sandstone, and discuss the relationship between permeability and volumetric strain [39]. Tan et al. constructed the constitutive relation of the permeability evolution law in fast Lagrangian analysis of continua 3D (FLAC3D) based on the Hoek-Brown failure criterion and simulated the complex hydro-mechanical coupling behavior during the progressive failure of low-porosity rocks [40].

In summary, researchers have explored the influence of seepage pressure on the physical and mechanical properties of rocks under the hydro-mechanical coupling by means of experiments and simulations, and found that the hydro-mechanical coupling of rocks is closely related to factors such as the development of fractures, the pore structure of rocks, and the physical properties of the fluid. However, there are still some shortcomings in the study of hydro-mechanical coupling. For example, in homogeneous sandstone reservoirs, the direction of crack propagation caused by hydro-mechanical coupling is difficult to control, which affects the distribution of seepage pressure in the rock mass and leads to an unsatisfactory permeability enhancement effect of the whole rock mass system. In order to deeply investigate the impact of fractures on directional damage of sandstone reservoirs and improve the efficiency of underground energy exploitation, this study conducts a hydro-mechanical coupling experiment considering T-shaped fractures.

The specific objectives of this article are: i. obtaining stress-strain curves and permeability variation curves of sandstone specimens with T-shaped fractures; analyzing the effect of fracture inclinations on mechanical properties such as the crack closure stress, crack initiation stress, strength, and axial strain stiffness of the specimens; and discussing the failure mode and permeability evolution process of T-shaped fracture samples. ii. According to damage mechanics, poroelastic medium theory and effective stress principle and combined with the test results, the Louis negative exponential seepage coupling equation is modified. ⅲ. The numerical simulation method is used to reproduce the rock failure process, and the distribution of seepage pressure and flow field vector field in the rock is analyzed, the fully coupled process of seepage-stress-damage is realized. The research results provide a scientific basis for the permeability enhancements technology in oil and gas and geothermal extraction engineering.

## 2. Test Method

### 2.1. Specimen Preparation

The sandstone selected for the test has no obvious texture on the surface; the material is hard in texture, light yellow in its natural state, and has a massive structure with a fine-medium granular sand-like structure. Specimens were prepared according to the standards of the International Society for Rock Mechanics. Cylindrical specimens with a diameter of 50 mm and a height of 100 mm were processed by coring, cutting, and grinding.

**Figure 1:**
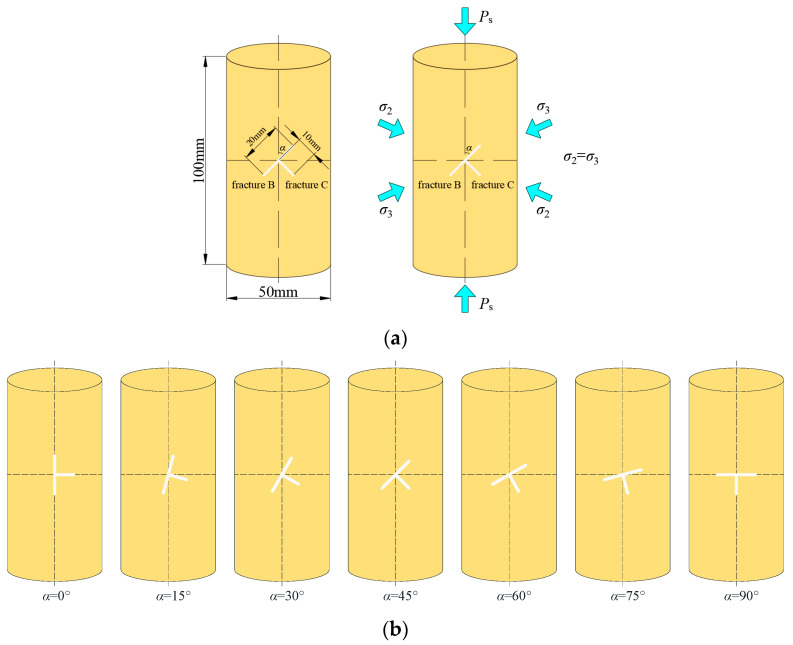
Schematic diagram of the T-shaped fractured sandstone specimen model: (**a**) Geometry and loading conditions, (**b**) Specimens with different fracture inclination angles.

Waterjet cutting and wire cutting are used to prefabricate T-shaped fracture sandstone specimens with different inclinations on cylindrical specimens (φ50 × 100 mm). The inclination is defined as the angle between the axial direction of the specimen and the clockwise rotation of the fracture. Each T-shaped fracture was 10 mm long and 0.3 mm wide. The geometry of the T-shaped fracture was characterized by the parameter α, and the values of α were 0°, 15°, 30°, 45°, 60°, 75°, and 90°, as shown in Figure 1.

### 2.2. Description of Rock Specimens

**Figure 2:**
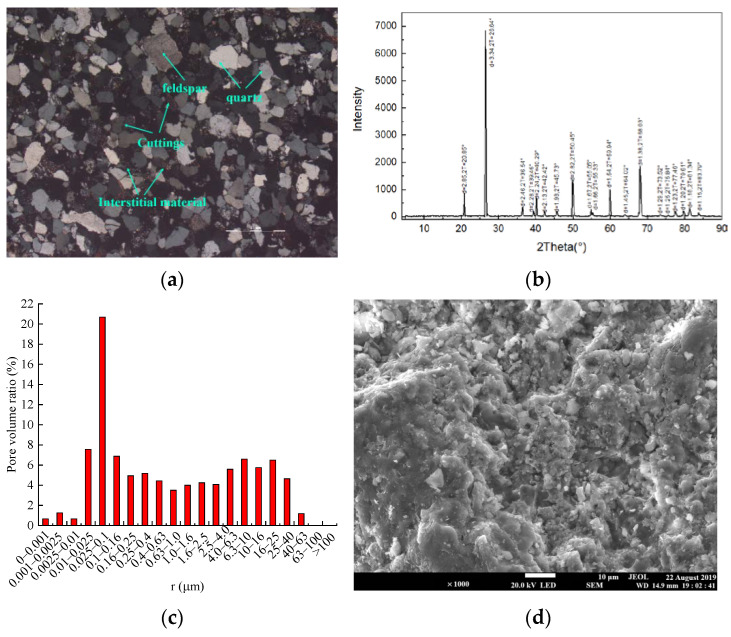
The detailed observation attempt of the specimen: (**a**) Structure observed by polarized light microscope, (**b**) X-ray diffraction (XRD) pattern, (**c**) Magnetic resonance imaging (MRI) pore radius map, (**d**) Scanning electron microscope (SEM) pattern.

The average density of 22 sandstone specimens was 2240.33 kg/m^3^, with an average longitudinal wave velocity of 2.19 km/s, and the homogeneity is good. In Figure 2, the mineral composition of sandstone obtained by polarizing microscope and X-ray diffraction analysis was 72.5% quartz, 5% feldspar, 12.5% debris, and 10% other. The average porosity obtained by the weighing method and nuclear magnetic resonance technology was 18.39%. At the same time, based on nuclear magnetic resonance technology, it can be seen that the pore size distribution was in the three intervals of 0~0.0025 μm, 0.0025~1 μm, and 1~63 μm, and the pore size was mainly distributed in the range of 0.001–40 µm, and the microscopic pore structure of sandstone was presented by scanning electron microscope (SEM).

### 2.3. Test Plan and Process

To study the mechanical characteristics and seepage law of T-shaped fracture sandstone specimens under the hydro-mechanical coupling, tests were carried out on T-shaped fracture sandstone specimens with different inclinations under the confining pressure of 10 MPa and the water pressure of 3 Mpa. In addition, the tests of two intact sandstone specimens are supplemented, which are intact specimens without water pressure (10 Mpa confining pressure) and intact specimens with water pressure (10 Mpa confining pressure and 3 Mpa water pressure), for comparative analysis. The specific test plan is shown in Table 1. The specimen number ST in the table represents the T-shaped fracture sandstone specimens, and the following numbers represent the inclinations, respectively. W1 represents an intact specimen without water pressure, and W2 represents an intact specimen with water pressure.

**Figure 3:**
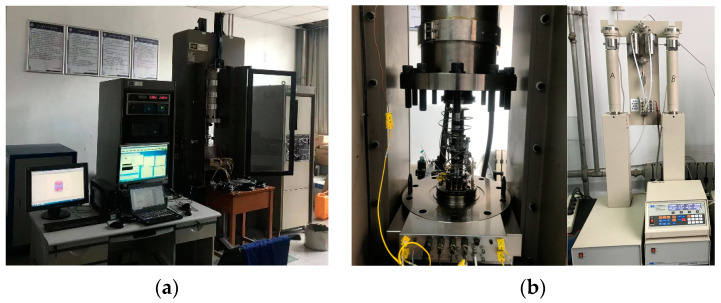
Hydro-mechanical coupling test equipment: (**a**) MTS815 rock mechanics testing machine, (**b**) Teledyne ISCO D-Series Pumps.

The test equipment adopted the MTS815 rock mechanics testing machine from American and the Teledyne ISCO D-Series Pumps hydraulic system, as shown in Figure 3. During the test, both the axial pressure and the confining pressure are controlled by the hydraulic servo system matched with MTS815, and the water pressure is controlled by the D-Series Pumps system. The deformation of sandstone specimens was measured using axial and hoop extensometers. For the reason that the prefabricated fracture of the specimen in the triaxial pressure chamber is easy to damage, the heat-shrinkable tube wrapping the specimen under the confining pressure results in the mixing of oil and water, which eventually leads to the failure of the test. Therefore, the gypsum mixed with water (gypsum quality:water quality = 2:1) was used to seal the front and back of the prefabricated fracture surface during the test, and the gypsum mixture on the surface of the fracture was allowed to solidify. The specimen is then wrapped with heat-shrinkable tubing to increase the compressive strength at the fracture of the specimen. During the test, the confining pressure was applied first, then the seepage pressure was applied, and the water pressure was always kept lower than the confining pressure. During axial loading, the entire loading process is controlled by a combination of load and deformation. In the initial loading stage, the axial load control method with a loading rate of 300 N/s was used for loading. When the load reached about 80% of the peak strength (55 Mpa), the loading method was switched to deformation control, and the loading rate was 0.02 mm/min until the rock broke.

The steady-state method can be used to measure the permeability [31]. The whole test process used pure water seepage, and the permeability was calculated based on Darcy’s law [32]. The calculation formula is as follows:

(1) $$K=-\frac{\mu QL}{A\Delta P}$$

where *K* is the permeability of the rock specimen, m^2^; *Q* is the volume flow through the specimen per unit time, m^3^/s; *μ* is dynamic viscosity coefficient of water, Pa · s; *L* is the height of the specimen, m; *A* is the cross-sectional area, m^2^, *A* = π*d*^2^/4; d is the diameter of the specimen; Δ*P* is the water pressure difference between the two ends of the specimen, Pa.

## 3. Numerical Simulation Method

The laboratory test mainly studies the mechanical properties of rock failure and the evolution law of permeability under hydro-mechanical coupling from a macroscopic perspective. To make up for the fact that the test cannot observe the progressive failure process and seepage track of the rock from a mesoscopic point of view, the RFPA2D-FLOW V2 software is used for numerical simulation. Numerical simulation research can be mutually verified and complemented with laboratory experiments. The process of numerical simulation is described in detail below.

### 3.1. Model Construction and Meshing

**Figure 4:**
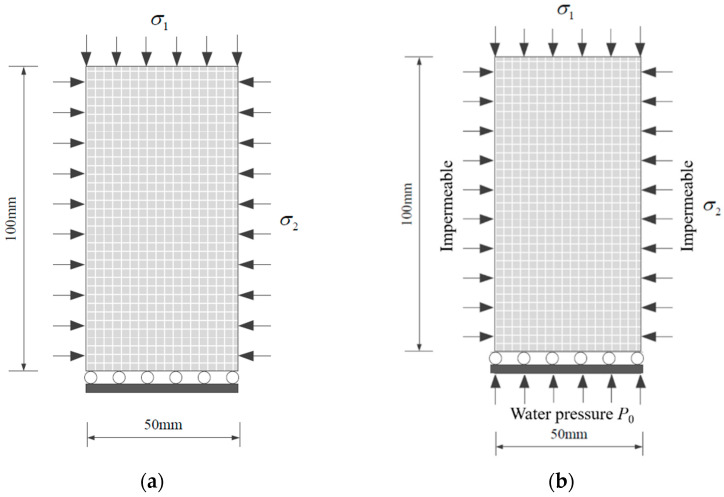
Numerical calculation model: (**a**) Conventional triaxial model, (**b**) Hydro-mechanical coupling model.

The geometric model of the conventional triaxial and hydro-mechanical coupling numerical tests is shown in Figure 4. The size of the model for the numerical test is 50 mm × 100 mm. Based on the model in Figure 4b, T-shaped fractures with different inclinations (0°~90°) are prefabricated, and the model grid is divided into 100 × 200. In order to characterize the heterogeneity of the sandstone, it is assumed that the initial mechanical parameters and seepage parameters of the sandstone specimen satisfy the Weibull distribution. The conventional triaxial and hydro-mechanical coupling simulation procedures are similar, with a constant 10 Mpa confining pressure applied on the left and right boundaries of the model and the bottom boundary being fixed. An axial load speed of 3 × 10^−5^ m/s was applied at the top boundary to control the load until the specimen completely lost its bearing capacity. In addition, the seepage behavior in the hydro-mechanical coupling test is simulated by the steady-state seepage model; the left and right boundaries of the specimen are impermeable boundaries, and water pressure of 3 Mpa is applied to the lower part of the specimen.

### 3.2. Governing Equations

The construction of the hydro-mechanical coupling model is based on the classical Biot consolidation theory [41] and the flow-stress-damage (FSD) coupling model established by Tang et al. and Yang et al. [42,43]. Biot’s consolidation theory has earlier studied the fluid-solid coupling problem, but this theory only considers the effect of seepage on soil consolidation and does not consider the effect of soil deformation on fluid seepage characteristics. The hydro-mechanical coupling effect is well considered by the FSD model, which explicitly expresses the relationship between stress, damage, and permeability. The specific hydro-mechanical coupling control equation is:

Equilibrium Equation:

(2) $$\sigma_{ij,j}+f_{i}=0\;\;\;\left(i,j=1,2,3\right)$$

Geometric equation:

(3) $$\varepsilon_{ij}=\frac{1}{2}\left(u_{i,j}+u_{j,i}\right)\;\;\;\left(i,j=1,2,3\right)$$

(4) $$\varepsilon_{v}=\varepsilon_{11}+\varepsilon_{22}+\varepsilon_{33}$$

Constitutive equation:

(5) $${\sigma^{\prime }}_{ij}=\sigma_{ij}-\alpha p\delta_{ij}=\lambda \delta_{ij}\varepsilon_{v}+2G\varepsilon_{ij}$$

Seepage equation:

(6) $$K\nabla^{2}p=\frac{1}{Q}\frac{\partial p}{\partial t}-\gamma \frac{\partial \varepsilon_{v}}{\partial t}$$

Coupling equation:

(7) $$K=\xi K_{0}e^{-\beta \left(\sigma_{ii}-\alpha p\right)}+C$$

where *σ_ij_* is the total stress tensor; *f_i_* is the component of the body force; *ε_ij_* is the strain tensor; *u_i,j_* are the displacement vectors; *ε_v_*, *ε_ii_* are the body strain and normal strain; ${\sigma^{\prime }}_{ij}$ is the effective stress tensor of the solid phase; *p* is the pore fluid pressure; *α* is the effective stress coefficient; *δ_ij_* is the Kronecker delta function; *γ* and *Q* are Biot constants; *G* and *λ* are shear modulus and Lame coefficient; $\nabla^{2}$ is Laplace operator; *σ_ii_* is average stress and *σ_ii_* = (*σ*_1_ + *σ*_2_ + *σ*_3_)/3; *β* is the coupling coefficient; and *ξ* is the penetration jump coefficient. The values of *C*, *β* and *ξ* are determined by the hydro-mechanical coupling test in this paper.

Equations (2)–(7) can not only reflect the influence of rock mass deformation characteristic parameters by pore water pressure but also reflect the change in permeability with the progressive failure process of cracks. The fractured rock mass will cause continuous changes in permeability in the process of progressive deformation and damage. When the stress on the fractured rock mass meets the critical damage threshold (the Mohr-Coulomb criterion is used for the strength criterion), the fractured rock mass begins to damage, the elastic modulus of damage is expressed as:

(8) $$E=\left(1-D\right)E_{0}$$

where *D* is the damage variable and *E* and *E*_0_ are the elastic modulus of the damaged rock mass and the non-damaged rock mass, respectively. Here, the damage to the rock mass is regarded as isotropic, so *D*, *E*, and *E*_0_ are all scalars.

Damage changes are often associated with sharp changes in permeability. Therefore, the seepage-damage coupling process can be described by the following equation. When the shear stress of the fractured rock mass reaches the Mohr-Coulomb damage threshold:

(9) $$F=\sigma_{1}-\sigma_{3}\frac{1+\sin\phi }{1-\sin\phi }\geq \sigma_{c}$$

where *σ*_1_ and *σ*_3_ are the maximum principal stress and minimum principal stress; *ϕ* is the friction angle; *σ*_c_ is the uniaxial compressive strength; and the damage variable *D* is expressed as follows:

(10) $$D=\left\{ \begin{array}{ll} 0 & \varepsilon <\varepsilon_{c0}\\ 1-\frac{\sigma_{cr}}{E_{0}\varepsilon } & \varepsilon_{c0}\leq \varepsilon \end{array}\right.$$

where *σ_cr_* is the residual strength, ε_c0_ is the compressive strain at the elastic limit, and the permeability of the fractured rock mass is described by a piecewise function:

(11) $$K=\left\{ \begin{array}{ll} K_{0}e^{-\beta \left(\sigma_{ii}-\alpha p\right)}+C & D=0\\ \xi K_{0}e^{-\beta \left(\sigma_{ii}-\alpha p\right)}+C & D>0 \end{array}\right.$$

When the fractured rock mass reaches the tensile strength *σ_t_*:

(12) $$\sigma_{3}\leq -\sigma_{t}$$

The expression of damage variable *D* is:

(13) $$D=\left\{ \begin{array}{ll} 0 & \varepsilon_{t0}\leq \varepsilon \\ 1-\frac{\sigma_{tr}}{E_{0}\varepsilon } & \varepsilon_{tu}\leq \varepsilon <\varepsilon_{t0}\\ 1 & \varepsilon \leq \varepsilon_{tu} \end{array}\right.$$

where *σ_tr_* is the residual strength; *ε_t_*_0_ is the strain at the elastic limit; and *ε_tu_* is the ultimate tensile strain of the rock mass, which describes the state that the rock mass will be completely damaged. The permeability of the fractured rock mass is also described by a piecewise function:

(14) $$K=\left\{ \begin{array}{ll} K_{0}e^{-\beta \left(\sigma_{ii}-\alpha p\right)}+C & D=0\\ \xi K_{0}e^{-\beta \left(\sigma_{ii}-\alpha p\right)}+C & D>0\\ \xi K_{0}e^{-\beta \left(\sigma_{ii}-p\right)}+C & D=1 \end{array}\right.$$

In summary, the core of the hydro-mechanical coupling control equation is the coupling equation. Based on the experimental results of the fractured sandstone specimen under the hydro-mechanical coupling, the key parameters in the coupling equation are obtained, which provide a basis for numerical simulation research. The mechanical parameters and seepage parameters are shown in Table 2.

## 4. Results and Discussion

### 4.1. Mechanical Properties of Fractured Sandstone UnderHydro-Mechanical Coupling

#### 4.1.1. Strength Properties

**Figure 5:**
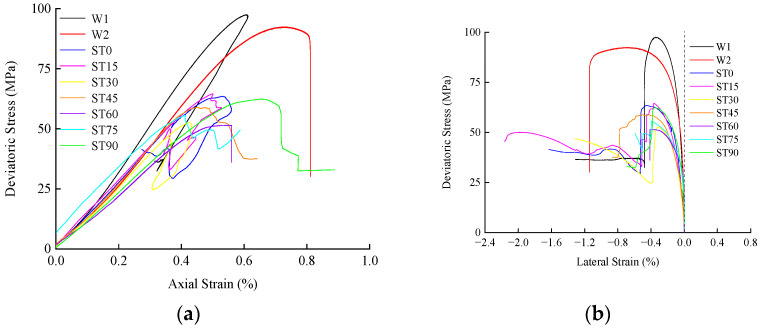
Stress-strain curves of intact and T-shaped sandstone specimens under hydro-mechanical coupling: (**a**) Axial strain-deviatoric stress relationship, (**b**) Lateral strain-deviatoric stress relationship.

When the confining pressure is 10 Mpa and the water pressure is 3 Mpa, the deviatoric stress-strain (axial strain, lateral strain) curves of the complete specimen and different angle T-shaped fracture specimens are shown in Figure 5. From the axial stress-strain curve, it can be seen that the curve has generally experienced the change process of nonlinear segment-linear segment-nonlinear segment, and the curve shows a concave trend at the initial stage of loading because, with the increase in load, the internal tiny pores are gradually closed (for hard rock, Zhao et al. believed that the reason for the concave curve may also be caused by the unevenness of the loading base [44]). And produce obvious nonlinear segments. With the increase in pressure, after the tiny pores are closed, the nonlinear segment disappears into a stable linear segment, and microcracks are formed locally in the specimen. It shows obvious nonlinear mechanical behavior before and after the peak. The post-peak stress-strain curve shows a vertical stress drop and a stepped stress drop to residual strength, both of which are obvious brittle failures. Comparing the intact specimen and the specimen with fractures, the peak value of the intact specimen is significantly larger than that of the specimen with fractures, and the peak value of the intact specimen without water pressure is greater than that of the intact specimen with water pressure, indicating that the existence of water pressure and fractures will significantly reduce the strength of the specimen.

**Figure 6:**
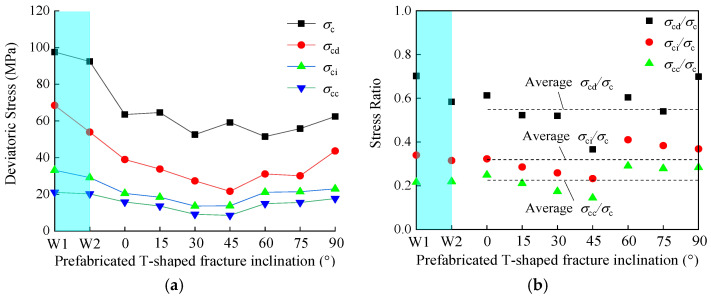
Relationship between fracture inclination and different thresholds, stress ratios of intact and T-shaped fracture samples: (**a**) Relationship between different thresholds and fracture inclination; (**b**) Relationship between stress ratio and fracture inclination.

The volumetric strain response method (VSR) proposed by Zhang et al. was used to obtain the crack closure stress σ_cc_, the crack initiation stress σ_ci_, and the damage stress σ_cd_, and the peak strength σ_c_ was extracted from the stress-strain curve [45]. The results of each key threshold are shown in Table 3 and Figure 6. It can be seen from the table that the key thresholds of different T-shaped fracture specimens under the hydro-mechanical coupling vary with the prefabricated fracture inclinations. It is found that the σ_c_ value is the smallest at 60° and the largest at 15°; the σ_cd_ value is the smallest at 45°and the largest at 90°; the σ_ci_ value is the smallest at 30° and the largest at 90°; the σ_cc_ value is the smallest at 45° and the largest at 90°. The average damage stress ratio σ_cd_/σ_c_ is 0.55, the average crack initiation stress ratio σ_ci_/σ_c_ is 0.32, and the average crack closure stress ratio σ_cc_/σ_c_ is 0.23 under different inclinations of the T-shaped specimen.

**Figure 7:**
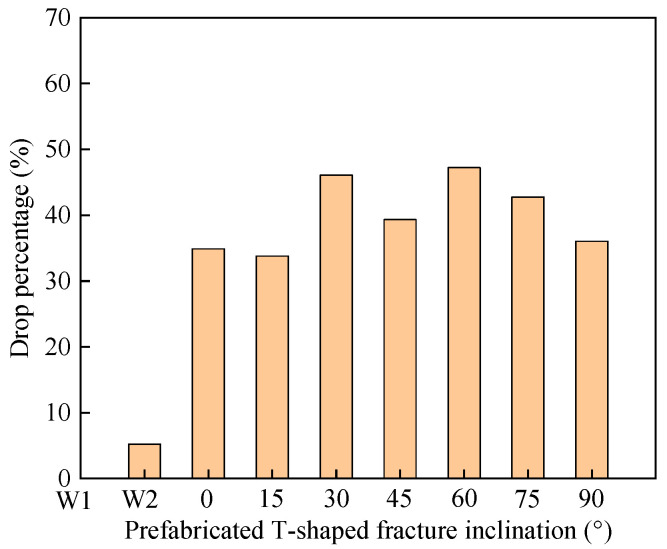
Drop Percentage in peak strength of intact specimens with water pressure, T-shaped fracture specimens compared to intact specimens without water pressure.

As shown in Figure 7, the peak strength of the intact specimen without water pressure is larger than the intact specimen with water pressure, which is greater than all fracture specimens. lower than that of the intact specimen with water pressure, the peak strengths of the T-shaped specimens decreased by 34.89%, 33.79%, 46.09%, 39.32%, 47.23%, 42.76%, and 36.02%, respectively, compared with the intact anhydrous specimens. It fully shows that the weakening effect of water has less influence on strength than prefabricated fractures. In addition, the stress ratios (crack closure stress ratio, crack initiation stress ratio, damage stress ratio) of the intact specimens without water pressure and intact specimens with water pressure are not significantly different from those of all the specimens with fractures, and the inclinations of the fractures have little effect on the stress ratios.

#### 4.1.2. Deformation Characteristics

**Figure 8:**
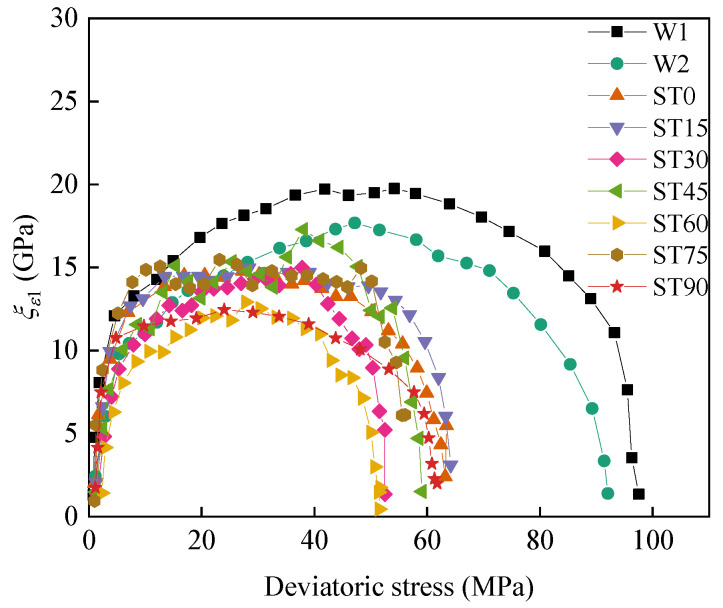
The relationship between axial strain stiffness and deviatoric stress under different angles T-shaped fracture sandstone.

In order to study the deformation characteristics of T-shaped fracture sandstone specimens under different fracture angles, the axial strain stiffness *ξ_ε_*_1_ was used to quantitatively characterize the ability of fractured sandstone to resist deformation and failure during the progressive failure process [16,19]. The formula for calculating *ξ_ε_*_1_ is as follows:

(15) $$\xi_{\varepsilon 1}\left(i\right)=\frac{1}{2}\left[\frac{\Delta \sigma_{1}\left(i\right)}{\Delta \varepsilon_{1}\left(i\right)}+\frac{\Delta \sigma_{1}\left(i-1\right)}{\Delta \varepsilon_{1}\left(i-1\right)}\right]$$

(16) $$\Delta \sigma_{1}\left(i\right)=\sigma_{1}\left(i+1\right)-\sigma_{1}\left(i\right)$$

(17) $$\Delta \varepsilon_{1}\left(i\right)=\varepsilon_{1}\left(i+1\right)-\varepsilon_{1}\left(i\right)$$

where *ξ_ε_*_1_(*i*) represents the axial strain stiffness at the *i*th data point of the stress-strain curve, GPa. Δ*σ*_1_(*i*) and Δ*ε*_1_(*i*) are the axial stress and strain increments, respectively. In addition, the increase in the axial deformation stiffness indicates that the ability of the rock to resist deformation and failure increases and conversely decreases. The relationship between the axial strain stiffness and deviatoric stress of the T-shaped fracture sandstone at different angles is shown in Figure 8. The axial strain stiffness in the figure increases sharply with the increase in deviatoric stress at the initial stage of loading, increases slowly before reaching the maximum axial strain stiffness, and finally decreases rapidly. The shape of the relationship between axial strain stiffness and deviatoric stress is less affected by the change in the inclinations of the prefabricated T-shaped fractures, but the intact specimen is affected by water flow and fractures. The maximum axial strain stiffness of the intact specimen without water pressure is greater than the intact specimen with water pressure, which is greater than all fracture specimens. It is also shown that water will weaken the ability to resist the deformation of sandstone, and the effect of water weakening on strength is less than that of prefabricated fractures.

#### 4.1.3. Failure Mode

**Figure 9:**
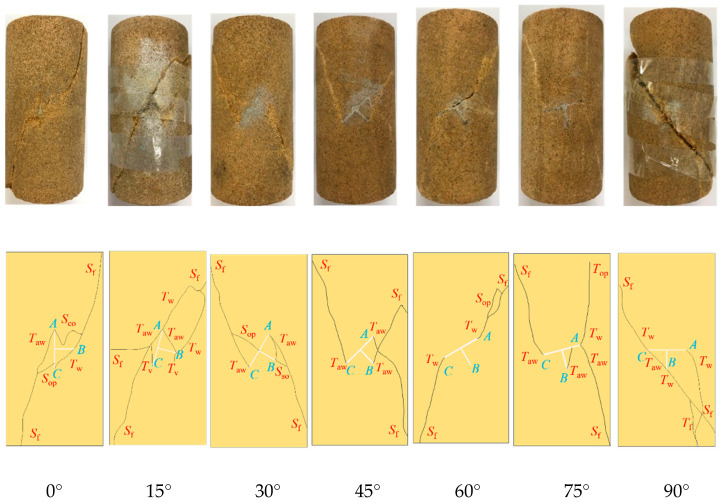
Failure mode of sandstone with T-shaped fractures.

The final fracture failure form of the T-shaped fracture sandstone specimen under the hydro-mechanical coupling is shown in Figure 9. It can be seen that the final fracture failure modes of the T-shaped fracture specimen are shear failure, tension failure, and tension-shear failure.

The specimen ST0 first cracks at the tips of cracks *A*, *B*, and *C*; the wing crack *T*_w_ expands toward the upper end, and the anti-wing crack *T*_aw_ expands toward the lower end. In addition, the coplanar secondary crack *S*_co_ generated at the tip of the crack *A* is connected with the wing crack *T*_w_ generated at the tip of the crack *B*, and then both are connected with the far-field shear crack *S*_f_ at the upper end. The far-field shear crack *S*_f_ at the lower end merges with the anti-wing crack *T*_aw_ and the out-of-plane shear crack *S*_op_ generated at the tip of the crack *A* and *C*.

When the fracture inclination reaches 15°, specimen ST15 experiences initial cracking at the tips of cracks *A* and *B*, and one wing crack *T*_w_ and two anti-wing cracks *T*_aw_ are generated at the tip of crack *A*; a wing crack *T*_w_, an anti-wing crack *T*_aw_, and a vertical tensile crack *T*_v_ are generated at the tip of crack *B*, and the anti-wing crack *T*_aw_ at crack *B* is coalesced and connected with the anti-wing crack *T*_aw_ at crack *A*. In addition, the wing crack *T*_w_ extending toward the upper end of the crack *A* and the crack *B* merge with the far-field shear crack *S*_f_ and penetrate the specimen. The vertical tensile crack *T*_v_ generated at the tip of the crack *C*, the anti-wing crack *T*_aw_ extending toward the lower end at *A*, and the two far-field shear cracks *S*_f_ merged and penetrated the specimen.

The specimen ST30 first fractures at the tips of cracks *A*, *B*, and *C* when the fracture angle is 30°. Two anti-wing cracks *T*_aw_ are generated at the tip of crack *A*, one of which connects to the anti-wing crack *T*_aw_ generated at the tip of crack *B*, and the other of which connects to the oblique secondary crack *S*_so_ generated at the tip of crack *B* and the far-field shear crack *S*_f_ at the lower end to merge and penetrate the specimen. The anti-wing crack *T*_aw_ generated at the tip of the crack *C* merges with the out-of-plane shear crack near the prefabricated crack and the far-field shear crack *S*_f_ at the upper end.

When the inclination of the fracture is 45°, the specimen ST45 first cracks at the tip of the cracks *A*, *B* and *C*, all of which produce reverse wing cracks *T*_aw_, which merge with the far-field shear cracks *S*_f_ at the upper and lower ends to cause the failure of the specimen.

The specimen ST60 initially fractures at the tips of cracks *A* and *C*, generating both wing cracks *T*_w_. The wing crack *T*_w_ that forms at the tips of crack *A* combines with the out-of-plane shear crack *S*_op_ and the far-field shear crack *S*_f_ at the upper end, while the wing crack *T*_w_ that forms at the tip of crack *C* directly connects with the far-field shear crack *S*_f_ at the lower end.

When the fracture inclination is at 75°, the specimen ST75 initially develops cracks at the tip of cracks *A*, *B*, and *C*. Subsequently, a wing crack *T*_w_ and an anti-wing crack *T*_aw_ are formed at the tip of crack *A*. The wing crack *T*_w_ propagates towards the upper end and connects to the out-of-plane tension crack *T*_op_, while the anti-wing crack *T*_aw_ extends towards the lower end and connects to the far-field shear crack *S*_f_. At the tip of crack *B*, an anti-wing crack *T*_aw_ is generated and linked to crack *A*. The anti-wing crack *T*_aw_ formed at the tip of crack *C* connects to the far-field shear crack *S*_f_.

Cracking initially occurs at the tips of cracks *A*, *B*, and *C* in specimen ST90. The wing crack *T*_w_ and the anti-wing crack *T*_aw_, formed at the tips of cracks *B* and *C*, respectively, combine and intersect with the far-field shear crack *S*_f_ at both the upper and lower ends, creating a shear crack. Additionally, the far-field tensile crack *T*_f_, which originates at the lower end, connects to the wing crack *T*_w_ that arises from cracks *A* and *B*.

In general, the final failure mode of T-shaped fracture specimens with different fracture inclinations is still dominated by shear failure under the hydro-mechanical coupling; when the inclinations of the fractures are 15°, 75° and 90°, a tensile-shear mixed failure occurs. In addition, some secondary cracks are accompanied by the failure process of the T-shaped fractures at different angles, and the final failure cracks *A* and *C* play the leading role.

### 4.2. Seepage Characteristics of Fractured Sandstone under the Hydro-Mechanical Coupling

**Figure 10:**
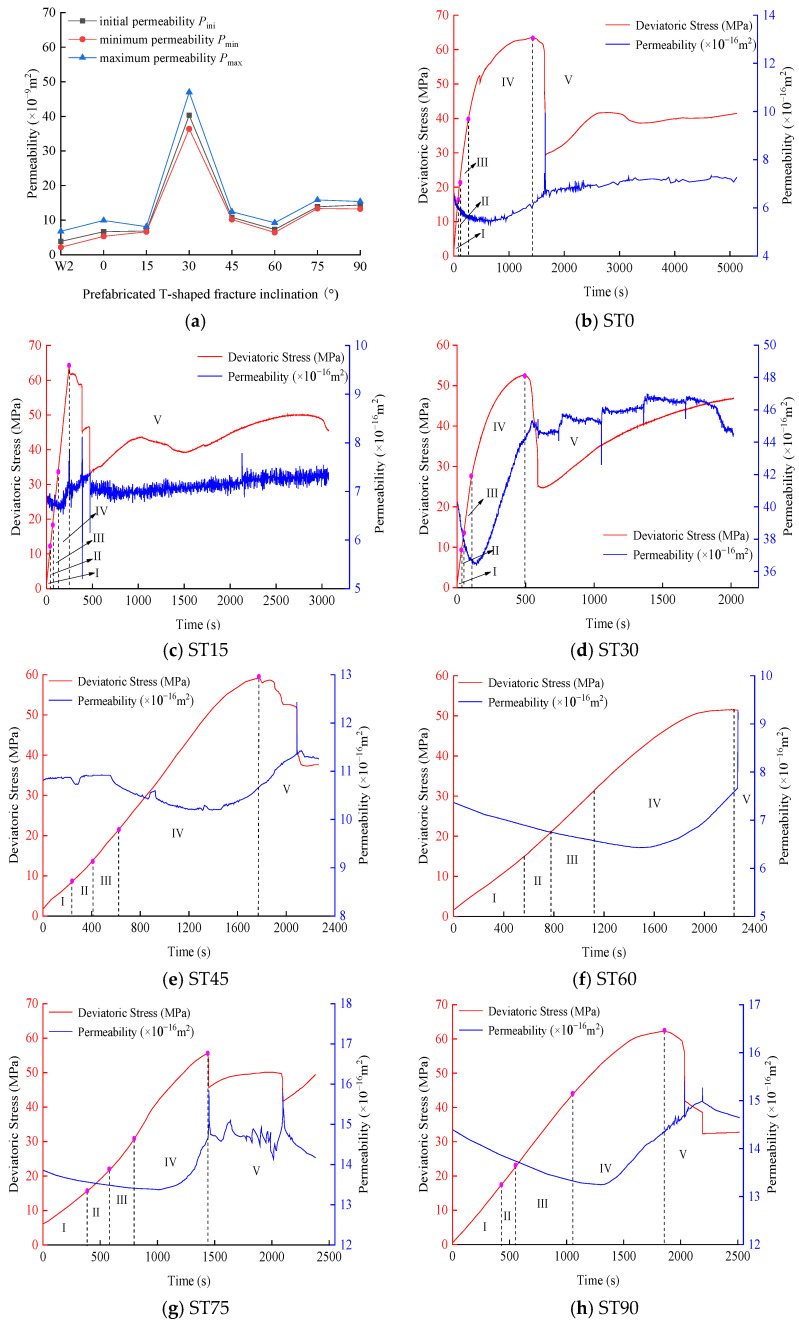
Variation of permeability characteristic points with different incliation fracture specimens:(**a**), Relationship between stress, permeability and time of T-shaped fracture specimen: (**b**–**h**).

**Figure 11:**
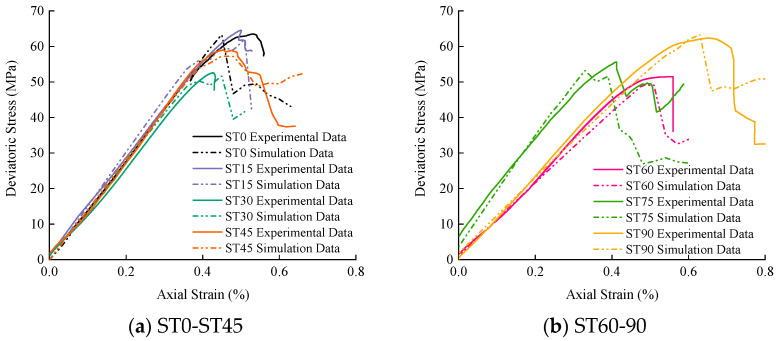
Comparison of test and simulation results of stress-strain curve of T-shaped fracture specimen.

Figure 10 shows the relationship between stress, permeability, and time of the T-shaped fracture specimen under the hydro-mechanical coupling. The curve is divided into five stages, namely: I—crack closure, II—elastic region, III—stable crack growth, IV—accelerated crack growth, and V—post-peak region. In the figure, from stage I to stage III, the permeability shows a decreasing trend, and the overall decrease in permeability in this stage is small; in stages IV and V, the rock volume changes from compression to expansion, and the rock volume strain continues expand, the permeability of rock first increases slowly, and then increases sharply with the drop of stress, the permeability of rock reaches a maximum value, and then decreases rapidly and tends to be stable as the fractures are compacted (stable increase, steady decrease, and approximate level). It can also be seen from Figure 11 that the maximum permeability occurs at the stress drop, not at the peak strength. In addition, the permeability of specimen ST30 is significantly higher than that of specimens with other angles. It is possible that the arrangement of microscopic pores and cracks in the specimen leads to better seepage channels, which eventually results in higher permeability. Based on the relationship between stress, permeability and time, the permeability characteristic points of sandstone specimens at different angles can be obtained, namely the initial permeability *P*_ini_, the minimum permeability *P*_min_ and the maximum permeability *P*_max_. At the same time, sudden jump coefficient is defined as the ratio of maximum to minimum permeability (*ξ* = *P*_max_/*P*_min_). Since the permeability characteristic point value is much larger than other angles at 30°, this point is abnormal and is not regarded as the maximum value. With the increase in the fracture inclination, the corresponding permeability characteristic points (*P*_ini_, *P*_min_ and *P*_max_) are the maximum values when the fracture inclination is 75°, which are 13.86 × 10^−16^ m^2^, 13.38 × 10^−16^ m^2^ and 15.84 × 10^−16^ m^2^, respectively. When the inclination of the fracture is 0°, the values of *P*_ini_ and *P*_min_ are the smallest, which are 6.67 × 10^−16^ m^2^ and 5.32 × 10^−16^ m^2^, respectively. When the inclination of the fracture is 15°, the *P*_max_ value is the smallest, which is 8.12 × 10^−16^ m^2^.

### 4.3. Verification of Numerical Results

#### 4.3.1. Progressive Failure Process of Fractured Sandstone

Based on the experimental plan, the progressive failure process and seepage evolution mechanism of fractured sandstone under hydro-mechanical coupling were deeply explored through numerical simulation. The comparison between the test and simulation results is shown in Figure 11. Combining with Table 4, it can be seen that the numerical simulation results are in good agreement with the laboratory test results; the variance and coefficient of variation between the peak strength and elastic modulus obtained by experiment and simulation are all within a reasonable range, indicating that the simulation results are reliable and reasonable.

**Figure 12:**
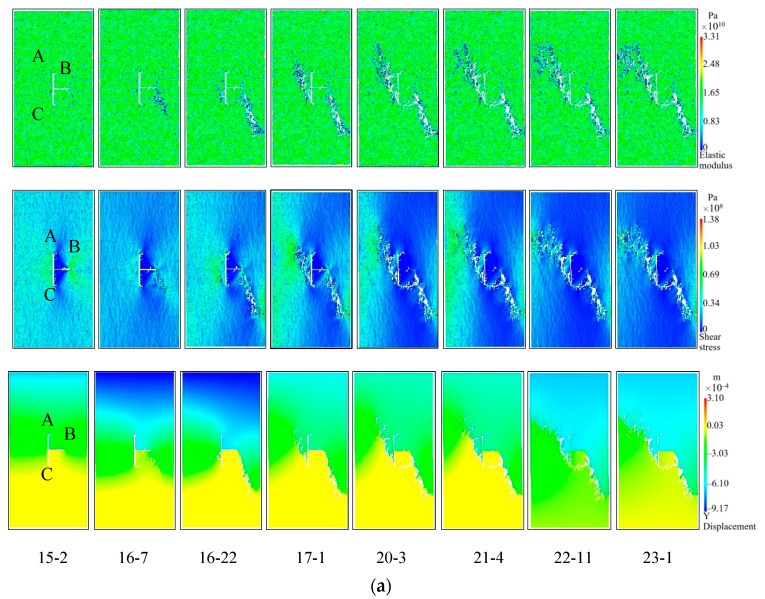

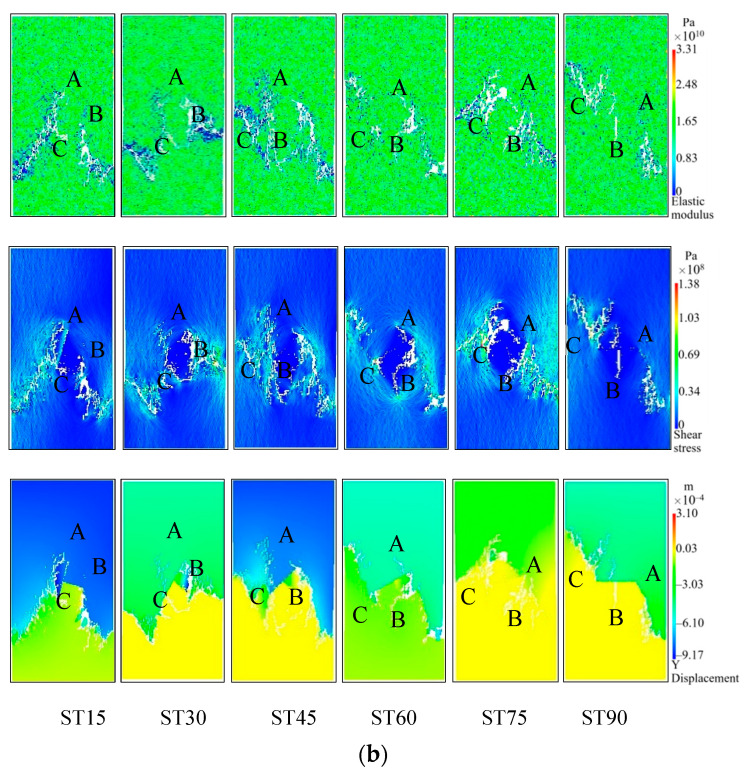
Simulation results of T-shaped fracture sandstone specimens under the action of hydro-mechanical coupling. (**a**) Progressive failure process of ST0 specimen, (**b**) Failure modes of ST15-ST90 specimens.

Figure 12 shows the simulation results of the failure process of the T-shaped fracture sandstone specimens under the hydro-mechanical coupling, since the RFPA2D-FLOW V2 software cannot characterize the initial compaction stage of the rock. Therefore, when analyzing the rock failure process in the simulation, stages I and II are regarded as linear elastic stages. Therefore, the stress-strain curve of the simulated specimen is divided into four stages by σ_ci_, σ_cd_, and σ_c_. In order to avoid redundancy, the case where the inclination of the T-shaped fracture specimen is 0° is taken as an example. The iteration step corresponding to σ_ci_ is 15-2 (“15” is the iteration step, “2” represents the second step in the 15 steps, that is, the step in the step), the iteration steps corresponding to σ_cd_ are 16-7, and the iteration steps corresponding to σ_c_ are 16-22. As shown in Figure 12a, with the increase in the load on the specimen ST0, when the iteration step is 15-2 and the axial displacement is 0.39mm, a new crack will start to form at the tip of the B crack, and because the shear stress is relatively concentrated, secondary cracks will appear. When the iteration step is 16-7, the axial displacement is 0.43mm, the stress concentration near the tip of the B crack is obvious, and micro-cracks are continuously generated at the weaker position at the lower right of the B crack, and the phenomenon of discontinuity and bifurcation occurs, gradually forming wing cracks. When the iteration step is 16-22 and the axial displacement is 0.45mm, the load reaches its peak strength, and the secondary crack extending downward from the tip of crack B to the lower right foot of the specimen finally forms a macroscopic through crack. At the same time, an anti-wing crack was formed at the tip of crack C, new cracks also appeared near the center of cracks A and C, and the macroscopic tensile crack started from the left tip of crack B. After the peak intensity, the calculation is continued until the iteration step is 23-1, at which time the axial displacement is 0.63mm. During this process, the secondary crack at the tip of crack C began to extend upward to the upper left of the specimen, and quickly penetrated with the edge of the model to form a macro crack, which eventually led to the failure of the model. Cracks B and C play a dominant role in the entire progressive failure process of the ST0 specimen. As shown in Figure 12b, the ST15-ST75 specimen prefabricated T-shaped fracture tip will produce wing cracks and anti-wing cracks, and only the ST90 prefabricated T-shaped crack tip will have wing cracks, and the T-shaped crack in the crack B tip produce fewer cracks, negligible compared with horizontal cracks, all specimens propagate in the direction of maximum principal stress. When approaching the peak strength, far-field shear cracks and secondary shear cracks are connected with wing cracks or anti-wing cracks, and gradually form main cracks and secondary shear crack bands, forming complex macroscopic fracture modes. After peak strength, a pronounced macroscopic crack forms and the specimen failure completely. In addition, the cracks A, C or B, C play the main control role in the whole progressive failure process of ST15-ST90 specimens, respectively, the displacement changes at the end of the simulation calculation are 0.66 mm, 0.65 mm, 0.67 mm, 0.63 mm, 0.69 mm, and 0.86 mm, respectively. The whole process of crack propagation and evolution is well presented by numerical simulation.

#### 4.3.2. Evolution Mechanism of T-Shaped Fracture Sandstone Permeability

**Figure 13:**
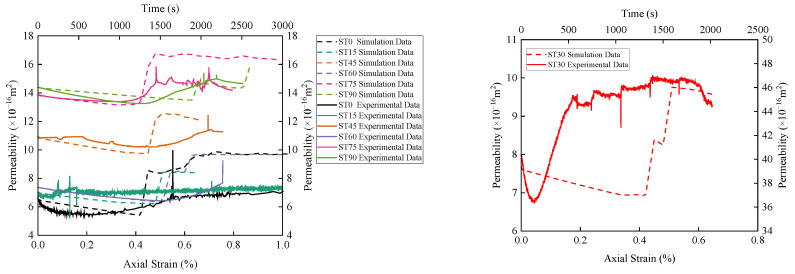
Comparison of permeability test and simulation results of ST0-ST90 specimens.

**Figure 14:**
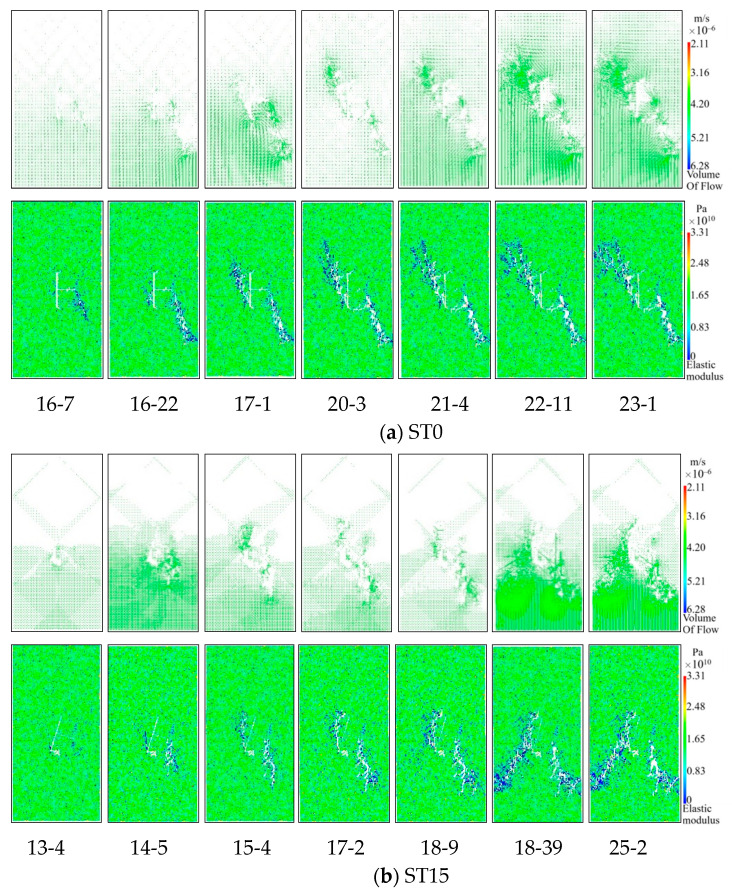

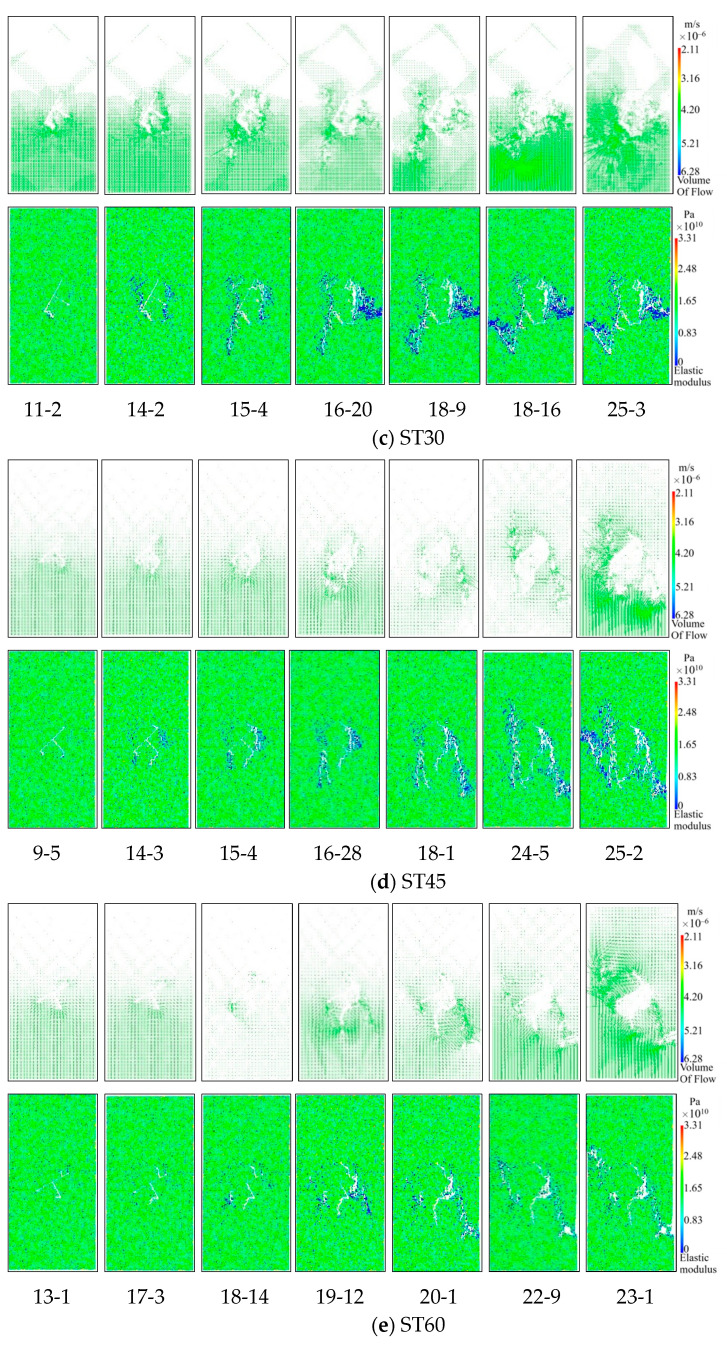

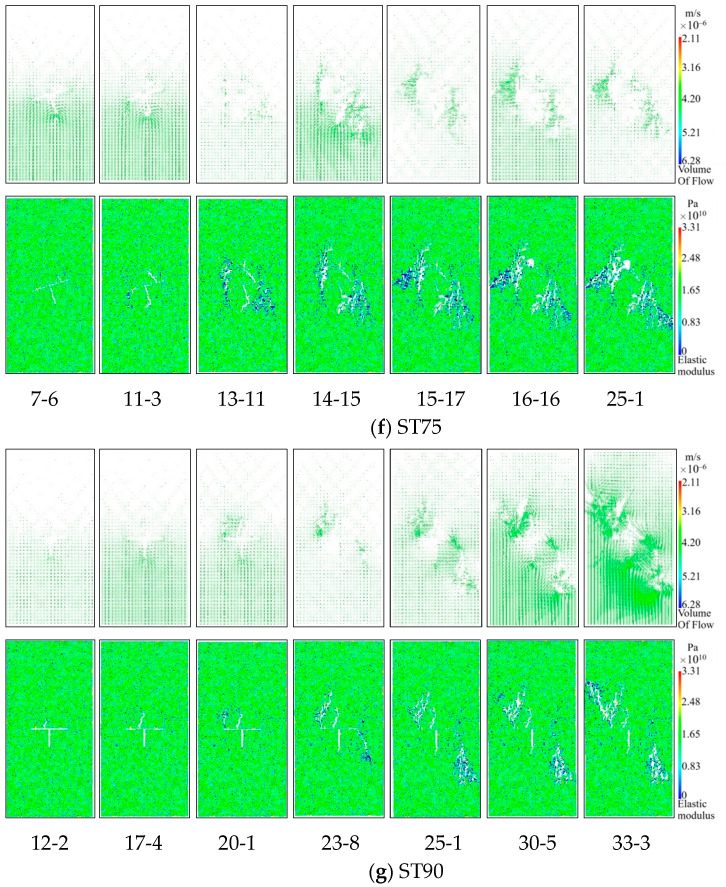
Flow vector and elastic modulus simulation results of T-shaped fracture sandstone specimens under hydro-mechanical coupling.

Taking *P*_min_ and *P*_max_ as critical points, the permeability change curve is divided into two stages, and the modified Louis negative exponential equation is used for fitting analysis. The key parameters in the seepage coupling Equation (7) are determined and numerically simulated. Figure 13 shows the simulation results of the relationship between the permeability and stress of the T-shaped fracture specimens. It can be seen from the figure that in the compaction and elastic stages of the microcracks, the pores in the specimen are compressed and the cracks are closed; as a result, the permeability shows a decreasing trend. With the increase in the axial stress, the microcracks enter the stage of stable expansion, and the internal cracks slowly increase in this stage, resulting in a slow decline of the permeability. In the stage of accelerated micro-crack expansion, the permeability Increases rapidly, and at the post-peak stage where the stress drops, the permeability begins to increase sharply, and a more obvious “sudden jump” phenomenon appears, which is in good agreement with the experimental results, indicating the feasibility of using the modified Louis negative exponential equation and the reliability of numerical simulation results. It can be seen from the flow vector and elastic modulus diagram in Figure 14 that the water flows in the direction of the formation of the main fractures. After the formation of the macroscopic main fractures, the flow Increases sharply and quickly occupies the entire main fracture channel. In addition, the permeability characteristic points obtained by numerical simulation and experiment are further compared and analyzed, as shown in Table 5. As can be seen from the table, except for specimen ST30 (the test data of this specimen is obviously larger than other specimens, it is abnormal data), the variance and coefficient of variation between the minimum permeability and the maximum permeability obtained by experiment and simulation are within a reasonable range, which further verifies the correctness of the simulation results.

## 5. Conclusions

In this paper, laboratory experiments are used to study the failure mechanism and permeability evolution law of T-shaped fracture rock under hydro-mechanical coupling, using the modified Louis negative exponential seepage coupling equation to simulate and reproduce the failure process of the rock and the distribution of the flow vector in the rock. The test results are compared with the simulation results and verified each other, and we reach the following conclusions:

(1) The crack closure, crack initiation, and damage stress ratios of the intact specimens without water pressure and the intact specimen with water pressure are not significantly different from those of all the fractured sandstone specimens, indicating that the stress ratios are hardly affected by the shapes and angles of the internal fractures of the rock. The propagation mode and deformation characteristics of T-shaped fractures are related to the flow parameters, and the weakening effect of water has less influence on the strength than prefabricated fractures. The hydro-mechanical coupling activates the prefabricated fractures and induces the expansion of T-shaped fractures to form a complex fracture network and increase the rock permeability. Tensile and shear failure modes formed by interconnected secondary fractures are the basic principles of permeability enhancement in sandstone reservoirs.
(2) Experiments have shown that there is a potential connection between T-shaped fractures and the hydraulic connectivity of rock discontinuities in rocks. Hydro-mechanical coupling action causes the cracks inside the rock to initiate first at the tip of the T-shaped fractures, and then with increasing coupled stress, the cracks propagate along the two main controlling cracks of the T-shaped fractures, forming different types of cracks, such as tensile cracks, shear cracks, coplanar secondary cracks, and oblique secondary cracks, eventually merging with the out-of-plane cracks and far-field tensile cracks.
(3) Under the axial load, the change in permeability of fractured rock is closely related to the development of fractures during the loading process. In the process of deformation and failure of T-shaped fracture specimens, due to the existence of cracks and the softening effect of water flow, the compaction stage to the stable crack propagation stage is shortened correspondingly. With the increase in axial pressure, the rock permeability first decreases and then increases in the pre-peak stage, and the sudden jump increases when reaching the strength failure. However, the maximum permeability occurs at the stress drop, not at the peak strength.
(4) Taking the shape and angle of the specimen into consideration, the average value of the sudden jump coefficient of the permeability of the T-shaped fracture specimen is 1.34. The simulated stress-strain curves and permeability evolution of sandstone are in good agreement with the experimental results. The modified seepage coupling model can better characterize the failure mechanism and seepage evolution of rocks under hydro-mechanical coupling. The flow vector distribution can intuitively reflect the effect of damage on permeability.

Based on the above research findings, it is helpful to better understand the hydraulic coupling behavior of fractured rock masses, which provides a scientific basis for the design of oil and gas wells and oil and gas and geothermal energy development and enhancement production. Besides, the findings provide a theoretical basis for the design and construction of hydraulic structures, such as tunnels and underground reservoirs, in fractured rock masses. At the same time, the research results have practical significance for the prediction and prevention of geological hazards associated with fractured rock masses, such as landslides and groundwater outbursts.

## Figures and Tables

**Table 1:** Test scheme of fractured and intact sandstone specimens.

Fracture Inclination α	Specimen No.		Confining Pressure/Mpa	Water Pressure/Mpa
	Intact Specimen	T-Shaped Fracture Specimen		
--	W1	--	10	--
--	W2	--	10	3
0°	--	ST0	10	3
15°	--	ST15	10	3
30°	--	ST30	10	3
45°	--	ST45	10	3
60°	--	ST60	10	3
75°	--	ST75	10	3
90°	--	ST90	10	3

**Table 2:** Hydro-mechanical coupling micromechanics and seepage parameters.

Fracture Inclinations/°	0	15	30	45	60	75	90
Elastic modulus *E*_0_/Gpa	17.38	17.88	16.25	17.00	14.13	20.50	14.86
Poisson’s ratio *μ*	0.26	0.31	0.31	0.43	0.09	0.29	0.15
Compressive strength *σ*_c_/Mpa	204.87	208.32	169.61	190.94	166.03	180.10	201.32
Friction angle *φ*/°	43	37	35	33	41	38	41
Permeability coefficient *k*/(m/d)	0.04	0.04	0.24	0.06	0.04	0.08	0.09
Effective stress factor *α*	0.80	0.38	0.29	0.29	0.41	0.87	0.33
Sudden jump coefficient *ξ*	1.87	1.22	1.29	1.22	1.44	1.18	1.16
Coupling coefficient *β*	0.0318	0.0850	0.0443	0.090	0.0251	0.0908	0.0139

**Table 3:** Summary of sandstone progressive failure thresholds under hydro-mechanical coupling.

Specimen No.	*σ*_cc_ (MPa)	*σ*_ci_ (MPa)	*σ*_cd_ (MPa)	*σ*_c_ (MPa)	*σ*_cc_/*σ*_c_	*σ*_ci_/*σ*_c_	*σ*_cd_/*σ*_c_
W1	17.300	32.630	68.480	97.540	0.216	0.340	0.702
W2	16.540	28.220	53.930	92.420	0.219	0.315	0.584
ST0	13.560	20.900	38.970	63.510	0.249	0.323	0.614
ST15	14.110	19.220	33.770	64.580	0.211	0.286	0.523
ST30	8.810	13.930	27.330	52.580	0.174	0.259	0.520
ST45	7.590	14.250	21.660	59.190	0.144	0.232	0.366
ST60	15.070	20.640	31.100	51.470	0.290	0.410	0.604
ST75	15.320	21.520	30.130	55.830	0.279	0.383	0.540
ST90	19.050	24.000	43.620	62.410	0.284	0.368	0.699

**Table 4:** Comparison of test and simulation results of mechanical properties of sandstone specimens.

Specimen No.	Test Strength *σ*_c_/Mpa	Simulation Strength *σ*_c0_/Mpa	SD	Cov/%	Test Elastic Modulus *E*/Gpa	Simulated Elastic Modulus *E*_0_/Gpa	SD	Cov/%
ST0	63.51	63.36	0.11	0.17%	13.90	15.52	1.15	7.79%
ST15	64.58	62.12	1.74	2.75%	14.30	17.66	2.38	14.87%
ST30	52.58	51.69	0.63	1.21%	13.00	15.47	1.75	12.27%
ST45	59.19	57.31	1.33	2.28%	13.60	15.76	1.53	10.40%
ST60	51.47	49.17	1.63	3.23%	11.30	10.79	0.36	3.27%
ST75	55.83	53.22	1.85	3.38%	16.40	17.68	0.91	5.31%
ST90	62.41	63.34	0.66	1.05%	11.89	11.87	0.01	0.12%

**Table 5:** Comparison of test and simulation results of the permeability characteristic points of sandstone specimens.

Specimen No.	Minimum Permeability/×10^−16^ m^2^		SD	Cov/%	Maximum Permeability/×10^−16^ m^2^		SD	Cov/%
	Test	Simulation			Test	Simulation		
W2	2.18	1.85	0.23	11.58%	6.80	6.87	0.05	0.72%
ST0	5.32	5.41	0.06	1.19%	9.93	10.21	0.20	1.97%
ST15	6.63	6.21	0.30	4.63%	8.12	8.46	0.24	2.90%
ST30	36.39	6.91	20.85	96.28%	46.97	9.75	26.32	92.80%
ST45	10.19	9.75	0.31	3.12%	12.43	12.56	0.09	0.74%
ST60	6.43	6.36	0.05	0.77%	9.24	9.72	0.34	3.58%
ST75	13.38	13.14	0.17	1.28%	15.84	16.76	0.65	3.99%
ST90	13.25	13.50	0.18	1.32%	15.41	16.05	0.45	2.88%

## Data Availability

Not applicable.

## References

[1] Gan Q., Elsworth D. (2014). Analysis of fluid injection-induced fault reactivation and seismic slip in geothermal reservoirs. J. Geophys. Res. Solid Earth.

[2] Wang H.L., Xu W.Y., Shao J.F. (2014). Experimental researches on hydro-mechanical properties of altered rock under confining pressures. Rock Mech. Rock Eng..

[3] Chen S.W., Yang C.H., Wang G.B. (2017). Evolution of thermal damage and permeability of Beishan granite. Appl. Therm. Eng..

[4] Huang N., Liu R.C., Jiang Y.J. (2017). Numerical study of the geometrical and hydraulic characteristics of 3D self-affine rough fractures during shear. J. Nat. Gas Sci. Eng..

[5] Rutqvist J., Tsang C.-F. (2002). A study of caprock hydromechanical changes associated with CO_2_-injection into a brine formation. Environ. Geol..

[6] Yuan S.C., Harrison J.P. (2006). A review of the state of the art in modelling progressive mechanical breakdown and associated fluid flow in intact heterogeneous rocks. Int. J. Rock Mech. Min..

[7] Jiang T., Shao J.F., Xu W.Y., Zhou C.B. (2010). Experimental investigation and micromechanical analysis of damage and permeability variation in brittle rocks. Int. J. Rock Mech. Min..

[8] Wang L., Liu J.-f., Pei J.-l., Xu H.-n., Bian Y. (2015). Mechanical and permeability characteristics of rock under hydro-mechanical coupling conditions. Environ. Earth Sci..

[9] Wanniarachchi W.A.M., Ranjith P.G., Perera M.S.A., Rathnaweera T.D., Zhang C., Zhang D.C. (2018). An integrated approach to simulate fracture permeability and flow characteristics using regenerated rock fracture from 3-D scanning: A numerical study. J. Nat. Gas Sci. Eng..

[10] Liu X., Lu W., Li M., Zeng N., Li T. (2020). The thermal effect on the physical properties and corresponding permeability evolution of the heat-treated sandstones. Geofluids.

[11] Lopes J.A., Medeiros W.E., Oliveira J.G., Santana F.L., Araújo R.E., La Bruna V., Xavier M.M., Bezerra F.H. (2023). Three-dimensional characterization of karstic dissolution zones, fracture networks, and lithostratigraphic interfaces using GPR cubes, core logs, and petrophysics: Implications for thief zones development in carbonate reservoirs. Mar. Pet. Geol..

[12] Wang D.-B., Zhou F.-J., Li Y.-P., Yu B., Martyushev D., Liu X.-F., Wang M., He C.-M., Han D.-X., Sun D.-L. (2022). Numerical simulation of fracture propagation in Russia carbonate reservoirs during refracturing. Pet. Sci..

[13] Medici G., West L.J. (2022). Review of groundwater flow and contaminant transport modelling approaches for the Sherwood Sandstone aquifer, UK; insights from analogous successions worldwide. Q. J. Eng. Geol. Hydrogeol..

[14] He R., Yang Z., Li X., Li Z., Liu Z., Chen F. (2019). A comprehensive approach for fracability evaluation in naturally fractured sandstone reservoirs based on analytical hierarchy process method. Energy Sci. Eng..

[15] Yang T.H., Liu J., Zhu W.C., Elsworth D., Tham L.G., Tang C.A. (2007). A coupled flow-stress-damage model for groundwater outbursts from an underlying aquifer into mining excavations. Int. J. Rock Mech. Min..

[16] Mitchell T.M., Faulkner D.R. (2008). Experimental measurements of permeability evolution during triaxial compression of initially intact crystalline rocks and implications for fluid flow in fault zones. J. Geophys. Res..

[17] Yang S.-Q., Huang Y.-H. (2020). Effect of damage on gas seepage behavior of sandstone specimens. J. Rock Mech. Geotech..

[18] Hou C., Ma J., Jin X., Ni D. (2020). Experimental study on the permeability evolution and gas flow law of post-strength soft coal. Appl. Sci..

[19] Zhou Y., Zhao D.J., Li B., Wang H., Tang Q., Zhang Z. (2021). Fatigue damage mechanism and deformation behaviour of granite under ultrahigh-frequency cyclic loading conditions. Rock Mech. Rock Eng..

[20] Kou M.M., Liu X.R., Wang Y.T. (2020). Study on rock fracture behavior under hydromechanical loading by 3-D digital reconstruction. Struct. Eng. Mech..

[21] Kou M.-M., Liu X.-R., Wang Z.Q., Nowruzpour M. (2021). Mechanical properties, failure behaviors and permeability evolution of fissured rock-like materials under coupled hydro-mechanical unloading. Eng. Fract. Mech..

[22] Baud P., Zhu W.L., Wong T.F. (2000). Failure mode and weakening effect of water on sandstone. J. Geophys. Res..

[23] Lajtai E.Z., Schmidtke R.H., Bielus L.P. (1987). The effect of water on the time-dependent deformation and fracture of a granite. Int. J. Rock Mech. Min. Sci. Geomech. Abstr..

[24] Masuda K. (2001). Effect of water on rock strength in a brittle regime. J. Struct. Geol..

[25] Helland J., Raab S. (2001). Experimental investigation of the differential stress on permeability of a lower Permian (Rotliegend) sandstone deformed in the brittle deformation field. Phys. Chem. Earth.

[26] Wang J.A., Park H.D. (2002). Fluid permeability of sedimentary rocks in a complete stress-strain process. Eng. Geol..

[27] Li P., Cai M.F., Gao Y.B., Wang P.T., Miao S.J., Wang Y. (2022). Fracture evolution and failure behavior around an opening in brittle jointed rocks subjected to uniaxial compression. Theor. Appl. Fract. Mech..

[28] Zhu W., Wong T.F. (1996). Permeability reduction in a dilating rock: Network modeling of damage and tortuosity. Geophys. Res. Lett..

[29] David C., Menendez B., Zhu W. (2001). Mechanical compaction, microstructures and permeability in sandstones. Phys. Chem. Earth.

[30] Chen Y.F., Hu S.H., Wei K., Hu R. (2014). Experimental characterization and micromechanical modeling of damage-induced permeability variation in Beishan granite. Int. J. Rock Mech. Min..

[31] Xiao W.J., Zhang D.M., Wang X.J. (2020). Experimental study on progressive failure process and permeability characteristics of red sandstone under seepage pressure. Eng. Geol..

[32] Lin Q., Cao P., Wang H., Cao R. (2018). An experimental study on cracking behavior of precracked sandstone specimens under seepage pressure. Adv. Civ. Engineering..

[33] Zhou H.W., Wang Z.H., Ren W.G., Liu Z.L. (2019). Acoustic emission based mechanical behaviors of Beishan granite under conventional triaxial compression and hydro-mechanical coupling tests. Int. J. Rock Mech. Min. Sci..

[34] Kou M., Liu X., Tang S., Wang Y. (2019). 3-D X-ray computed tomography on failure characteristics of rock-like materials under coupled hydro-mechanical loading. Theor. Appl. Fract. Mech..

[35] Akhondzadeh H., Keshavarz A., Awan F.U.R., Al-Yaseri A.Z., Iglauer S., Lebedev M. (2020). Coal fracturing through liquid nitrogen treatment: A micro-computed tomography study. APPEA J..

[36] Akhondzadeh H., Keshavarz A., Awan F.U.R., Ali M. (2021). Liquid nitrogen fracturing efficiency as a function of coal rank: A multi-scale tomographic study. J. Nat. Gas Sci. Eng..

[37] Yuan S.C., Harrison J.P. (2005). Development of a hydro-mechanical local degradation approach and its application to modelling fluid flow during progressive fracturing of heterogeneous rocks. Int. J. Rock Mech. Min..

[38] Cai Y.Y., Chen X., Yu J., Zhou J. (2018). Numerical study on the evolution of mesoscopic properties and permeability in sandstone under hydro-mechanical coupling conditions involving industrial internet of things. IEEE Access.

[39] Chen X., Yu J., Tang C., Li H., Wang S. (2017). Experimental and numerical investigation of permeability evolution with damage of sandstone under triaxial compression. Rock Mech. Rock Eng..

[40] Tan X., Konietzky H., Frühwirt T. (2014). Laboratory observation and numerical simulation of permeability evolution during progressive failure of brittle rocks. Int. J. Rock Mech. Min..

[41] Biot M.A. (1941). General Theory of Three-Dimensional Consolidation. J. Appl. Phys..

[42] Tang C.A., Tham G., Lee P.K.K., Yang T.H., Li L.C. (2002). Coupled analysis of flow, stress and damage (FSD) in rock failure. Int. J. Rock Mech. Min. Sci..

[43] Li P., Cai M.F., Gao Y.B., Gorjian M., Miao S.J., Wang Y. (2022). Macro/mesofracture and instability behaviors of jointed rocks containing a cavity under uniaxial compression using AE and DIC techniques. Theor. Appl. Fract. Mech..

[44] Zhao X.G., Cai M., Wang J., Ma L.K. (2013). Damage stress and acoustic emission characteristics of the Beishan granite. Int. J. Rock Mech. Min. Sci..

[45] Zhang Y., Wu X., Guo Q., Zhang Z., Cai M., Tian L. (2022). Research on the Mechanical Properties and Damage Constitutive Model of Multi-Shape Fractured Sandstone under Hydro-Mechanical Coupling. Minerals.

